# ResRandSVM: Hybrid Approach for Acute Lymphocytic Leukemia Classification in Blood Smear Images

**DOI:** 10.3390/diagnostics13122121

**Published:** 2023-06-20

**Authors:** Adel Sulaiman, Swapandeep Kaur, Sheifali Gupta, Hani Alshahrani, Mana Saleh Al Reshan, Sultan Alyami, Asadullah Shaikh

**Affiliations:** 1 Department of Computer Science, College of Computer Science and Information Systems, Najran University, Najran 61441, Saudi Arabia; aaalsulaiman@nu.edu.sa (A.S.); hmalshahrani@nu.edu.sa (H.A.); 2 Chitkara University Institute of Engineering and Technology, Chitkara University, Rajpura 140401, Punjab, India; swapandeep.kaur@chitkara.edu.in (S.K.); sheifali.gupta@chitkara.edu.in (S.G.); 3 Department of Information Systems, College of Computer Science and Information Systems, Najran University, Najran 61441, Saudi Arabia; msalreshan@nu.edu.sa (M.S.A.R.); asshaikh@nu.edu.sa (A.S.)

**Keywords:** acute lymphocytic leukemia, ResNet152, VGG16, DenseNet121, MobileNetV2, InceptionV3, EfficientNetB0, ResNet50, analysis of variance, principal component analysis, deep learning, machine learning, naïve Bayes, random forest, artificial neural network, adaboost, support vector machine

## Abstract

Acute Lymphocytic Leukemia is a type of cancer that occurs when abnormal white blood cells are produced in the bone marrow which do not function properly, crowding out healthy cells and weakening the immunity of the body and thus its ability to resist infections. It spreads quickly in children’s bodies, and if not treated promptly it may lead to death. The manual detection of this disease is a tedious and slow task. Machine learning and deep learning techniques are faster than manual detection and more accurate. In this paper, a deep feature selection-based approach ResRandSVM is proposed for the detection of Acute Lymphocytic Leukemia in blood smear images. The proposed approach uses seven deep-learning models: ResNet152, VGG16, DenseNet121, MobileNetV2, InceptionV3, EfficientNetB0 and ResNet50 for deep feature extraction from blood smear images. After that, three feature selection methods are used to extract valuable and important features: analysis of variance (ANOVA), principal component analysis (PCA), and Random Forest. Then the selected feature map is fed to four different classifiers, Adaboost, Support Vector Machine, Artificial Neural Network and Naïve Bayes models, to classify the images into leukemia and normal images. The model performs best with a combination of ResNet50 as a feature extractor, Random Forest as feature selection and Support Vector Machine as a classifier with an accuracy of 0.900, precision of 0.902, recall of 0.957 and F1-score of 0.929.

## 1. Introduction

Cancer is the most deadly disease and has a negative impact on people all over the world. Among all types of cancer, blood cancer is the most dangerous in its later stages. The disease related to white blood cells (WBC) is known as leukemia. WBCs, also known as leukocytes, are one of the blood constituents and constitute one percent of the total blood volume. Human immunity is dependent on WBCs. The other blood constituents include red blood cells (RBCs) and platelets. Leukocytes, or WBCs, help fight infections and diseases. Leukemia is a cancer that destroys human immunity by affecting the bone marrow.

Leukemia leads to the production of immature leukocytes in large numbers. Leukemia is further divided into two types, chronic and acute. If the disease increases rapidly, it is acute leukemia; when it grows slowly, it is chronic leukemia. The symptoms of acute leukemia are more severe than chronic leukemia. Acute Lymphocytic Leukemia (ALL) [1] is a type of WBC cancer caused by consistent multiplication and unrestrained production of immature leukocytes in the bone marrow. ALL is predominantly found in children, constituting about 25% of all cancers in children. This cancer has similar symptoms to those of the common flu and other symptoms such as weakness, joint pains, fatigue, etc., making diagnosing this disease very difficult. This disease poses a significant risk to one’s life. The survival time of ALL patients is 3 months only if treatment is not given on time. Hence, appropriate treatment and therapy are vital for saving the patient’s life.

The manual detection of this cancer requires an expert doctor or physician for early and accurate detection. The examination of blood smear images has become common for the detection of ALL. However, manual detection has problems such as noise, blur, weak edges and the complex nature of blood cells, and is also reliant on human interpretation. Machine learning (ML) and deep learning (DL) advancements can help in detecting the disease more accurately and also help doctors to diagnose and treat the condition properly [2]. The procedure adopted includes pre-processing the images, feature extraction, feature selection, and classification.

ML techniques [3] are also gaining importance in the classification of mitosis in breast cancer. Rehman et al. [4] constructed a novel model that involved neural-network-based [5] concepts and ML classifiers for the classification of mitotic and non-mitotic cells. The cell texture was used for deriving reduced feature vectors through multiple techniques and classification by ML classifiers such as SVM, Random Forest and Naïve Bayes. The ML classifiers performed better than the neural networks for this breast cancer dataset. Neural networks require more time to process the images and the images are also insufficient for optimum training of the neural network. Furthermore, the images are small, which leads to overfitting of the model. Hence, in this paper a hybrid model is used, which includes the best combination of both DL and ML techniques.

The significant contributions of this research paper are:

- A deep feature selection-based approach is proposed for detecting Acute Lymphocytic Leukemia in which ResNet152, VGG16, DenseNet121, MobileNetV2, InceptionV3, EfficientNetB0 and ResNet50 are used as feature encoding networks to extract deep features from blood smear images.
- From the deep feature pool, valuable and significant features are extracted using ANOVA, PCA and Random Forest feature selection methods.
- The selected top features are classified using ensemble voting of four classifiers, Adaboost, Naïve Bayes, Artificial Neural Network and Support Vector Machine, to classify the images into ALL and normal classes. The performance of the proposed approach is measured in terms of accuracy, precision, recall, and F1-score.

The remaining portion of this paper is divided into a literature review in Section 2, a proposed deep feature selection-based approach in Section 3, comparison with state-of-art models Section 4, and a conclusion and summary of future scope in Section 5.

## 2. Literature Review

Leukemia is a group of blood cancers affecting bone marrow and blood cells. It is a complex and heterogeneous disease that requires accurate diagnosis, classification, and treatment. ML has shown great potential in enhancing the accuracy and efficiency of leukemia diagnosis and classification and predicting treatment outcomes. Jagadev et al. [6] compared the performance of several machine-learning algorithms for leukemia classification using gene expression data. The results showed that support vector machines (SVM) outperformed other algorithms in terms of accuracy and speed. Ratley et al. [7] put forward a hybrid ML system for the detection and further classification of leukemia images. This approach used a combination of convolutional neural networks (CNN) and an SVM classifier for achievement of high accuracy. Lee et al. [8] proposed a ML model for prediction of the extent of sensitivity of drugs. The model helped in accurate prediction of responses to drugs and also identified potential drug targets. Saeed et al. [9] put forward a DL-based model for the diagnosis of leukemia using blood cell images. The images were classified into normal and leukemia images using a transfer learning CNN model with high accuracy. In the subsequent study, Shaikh et al. [10] developed a machine-learning model to predict the survival of acute myeloid leukemia patients using clinical and genetic data. The model accurately predicted patient outcomes and identified potential prognostic biomarkers.

The SVM classifier is commonly used for leukemia classification as it gives high accuracy and a linearly separable feature space is not required. The SVM classifier works well with both semi-structured as well as unstructured data [11]. It is one of the most efficient ML techniques. It can handle large feature spaces and non-linear feature interactions which do not rely on the entire dataset [12].

Chen et al. [13] proposed a label augmented and weighted majority voting (LAWMV) model for crowdsourcing purposes. This model outperformed other state-of-the-art models by achieving an accuracy of 82.89%. Majority voting is a simple and an effective method for integration [14]. Rehman et al. [4] developed a m6A-Neural Tool for the prediction and identification of m6A sites. This model used majority voting on three sub-architectures. These architectures used a set of convolutional layers to extract the important features from the input. An increased accuracy was obtained by this model as compared to other existing models. It achieved an accuracy of 93.9% for A. thaliana species, 91.5% for M. musculus and 92% accuracy for H. sapiens species. Singh et al. [15] introduced a hybrid system to classify images of skin affected by lesions. The model was compared with commonly used techniques. The hybrid model utilized majority voting and principal component analysis and factor analysis and achieved an accuracy of 96.80%.

Finally, from the above literature, it can be inferred that ML has great potential to help in the diagnosis and classification of leukemia. This would further help to treat leukemia on a timely basis. However, further research needs to be carried out for the validation of the above models in clinics and hence integrate them in leukemia care on a routine basis.

DL is a subset of artificial intelligence that utilizes neural networks for the analysis and interpretation of data. DL is now gaining increased interest for improvement in leukemia diagnosis, classification, identification and treatment. Boldu et al. [16] proposed a DL system for the classification of acute myeloid leukemia (AML) using blood smear images. This system attained a very good accuracy of 96.4%, thus outperforming other ML techniques. It also exhibited how well DL techniques can help to predict leukemia. Bodzas et al. [17] implemented with a DL model for diagnosing ALL using blood smear images and obtained an accuracy of 94.8%. Boldu et al. [16] created a DL model for classification of leukemia subtypes and achieved an accuracy of 91.7%. Regarding the testing time for a single blood cell image, the BCNet model is proposed. This model outperformed the AI models of DenseNet, ResNet, Inception, and MobileNet by 10.98, 4.26, 2.03, and 0.21 msec. The BCNet model may produce positive results compared to the most recent deep learning algorithms [18]. In a different paper, El Achi et al. [19] put forward a DL model to classify lymphoma subtypes and attained an accuracy of 97.7%. This paper exhibited how well the DL model can improve lymphoma diagnosis. Islam et al. [20] developed a DL model for predicting how well the patient recovers after giving chemotherapy to patients suffering with AML. This model attained an accuracy of 86.3%. So, it can be seen that DL models perform quite well for prediction of leukemia. Hence, both the ML and DL techniques can be integrated to achieve good results in classification of leukemia. In this paper, because of this reason, the author has used a hybrid model that integrates both DL and ML techniques.

## 3. Proposed Deep Feature Selection Based Approach

**Figure 1:**
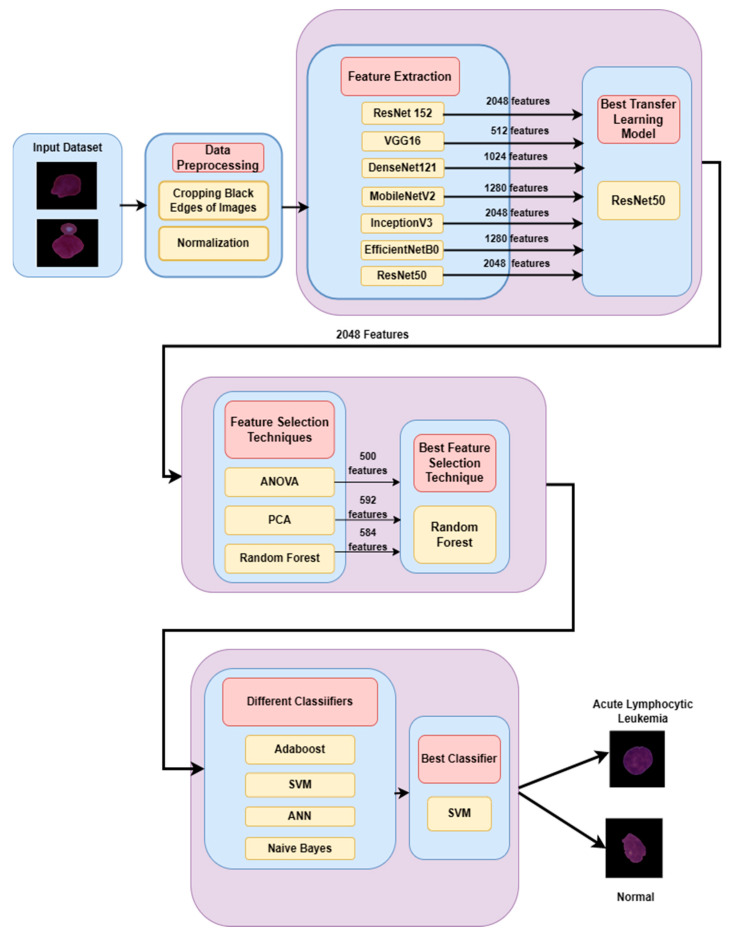
ResRandSVM: Proposed approach for leukemia detection in blood smear images.

Figure 1 shows the proposed deep feature selection-based approach for the detection and classification of leukemia. The pre-processing of the input dataset is carried out by cropping the black edges of the images. After that, the normalization of images is performed to converge faster during training by reducing the impact of large input value ranges that may cause vanishing gradients. Feature extraction was conducted through ResNet152, VGG16, DenseNet121, MobileNetV2, InceptionV3, EfficientNetB0, and ResNet50 models. The best feature extraction model was ResNet50, which extracted 2048 features. Feature selection was carried out using three feature selection methods: ANOVA, PCA, and Random Forest. Random Forest was the best feature selection technique because it selected 584 features from a pool of 2048 features. Finally, the classification of the images has carried out using four classifiers, i.e., Adaboost, SVM, ANN, and Naïve Bayes.

The best results were achieved by combining the Resnet50 model, which performs feature extraction, the random forest model, which is used for feature selection, and the SVM model, which performs the classification of the images into leukemia and normal image classes. The novelty of the ResNet-50 (feature extraction)–Random Forest (feature selection)–SVM (classification) hybrid approach lies in the combination of these three components used to create a comprehensive pipeline that addresses the image classification task.

ResNet-50 is a deep CNN architecture that excels at extracting high-level and hierarchical features from images. By leveraging the pre-trained ResNet-50 model, the hybrid model can extract meaningful representations from the input images. These extracted features capture complex patterns and enable more effective discrimination between different classes.

Random Forest is an ensemble technique and is a combination of several decision trees. In this hybrid model, the Random Forest classifier has been used as a feature selector. The Random Forest model assesses the importance of each feature and ranks them accordingly. Hence, it has been used as a feature selector after the ResNet5 feature extraction technique. The Random Forest feature selection method gives the most essential features as outputs, which further are provided to the classification stage.

SVM is a popular classifier that learns the decision boundary between different classes on the basis of the selected features, leading to accurate classification. Hence, SVM has been used for classification after the feature selection step.

Thus, this hybrid model combination works on the strengths of DL-based feature extraction, Random Forest feature selection and classification through SVM.

### 3.1. Input Dataset

**Figure 2:**
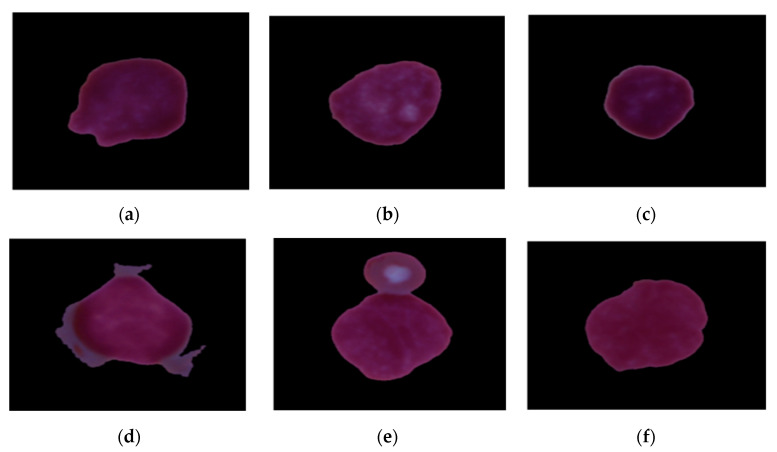
Sample images. (**a**–**c**) Healthy WBCs. (**d**–**f**) Leukemia-affected WBCs.

The dataset was obtained from Kaggle (CNMC 2019) [21]. The dataset comprises 10,661 images, out of which 7272 are of patients suffering from leukemia and 3389 images are of patients who are not suffering from leukemia, as shown in Figure 2. The images were divided into the training set and test set, in which 20% of images are of the test set; that is, there are 2133 test images and 8528 training images. Figure 2a–c shows the healthy WBCs, and Figure 2d–f shows the WBCs affected by leukemia.

### 3.2. Data Preprocessing

The primary step in image processing [22] is the pre-processing stage, as it leads to an enhancement in the characteristics of the leukemia images. It also suppresses the noise and unwanted data prevalent in the images. Cropping the black edges and normalization are the two steps in data pre-processing.

#### 3.2.1. Cropping Black Edges in Images

**Figure 3:**
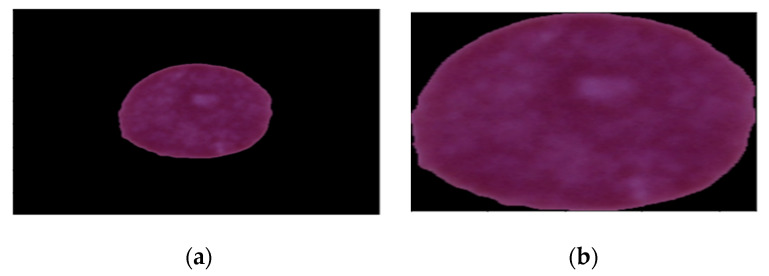
Sample images. (**a**) Before cropping. (**b**) After cropping.

Cropping black edges in images can be completed using various image processing techniques. The first step involves the conversion of the images to grayscale images. This step makes the detection of edges easier because the images are reduced to a single channel. The next step is to detect the edges of the images. The final step is cropping the image using the coordinates of the bounding box. This is done using the OpenCV’s crop function. Hence, automatic cropping of the black edges present in an image can be carried out using these steps. Figure 3a shows the image before cropping and (b) shows the image after cropping.

#### 3.2.2. Normalization

Normalization is a data preprocessing technique commonly used to rescale the numerical features of a dataset so that they have similar scales and ranges. Normalization is important because many machine learning algorithms perform best when the normalization of the input is done. For example, algorithms that are based on distance metrics, such as KNN or SVM, are more sensitive to the scale of the input features.

Normalization can be carried out in several ways, but the most common method includes min-max scaling. This method scales the features so that they have the least value of 0 and the greatest value of 1 [23,24].

Normalization is applied to the training data before training, and then the same scaling factors are applied to the test data. This step is required in order to maintain numerical stability in the DL models. Normalization makes the learning quicker and also increases the stability of the gradient descent. The pixel values are normalized in the range of 0–1 which is obtained by multiplying 1/255 with the pixel values.

### 3.3. Feature Extraction Using Different Transfer Learning Models

Transfer learning involves applying an already-learned model to a new problem. In transfer learning, knowledge gained from training a model on one task is used to improve performance on a different but related task. In this paper, six transfer learning models, ResNet152, VGG16, DenseNet121, MobileNetV2, InceptionV3, and EfficientNetB0, which are pre-trained models, were trained on a large dataset and used for feature extraction. The results for each transfer learning model are given in the subsequent sections.

#### 3.3.1. Feature Extraction Using ResNet152

ResNet-152 [25] is a deep CNN architecture with a total of 152 layers that addresses the issue of vanishing gradients in deeper models. ResNet152 introduces residual connections that are also known as skip connections. These connections help overcome degradation problems that occur in deep networks. The ResNet152 model comprises various residual blocks that include basic blocks with two convolutional layers and bottleneck blocks with three convolutional layers. The bottleneck blocks help reduce the computational cost. ResNet152 model also introduces the concept of identity mapping, in which input given to a residual block is directly connected to the output, bypassing the convolutional layers.

**Figure 4:**
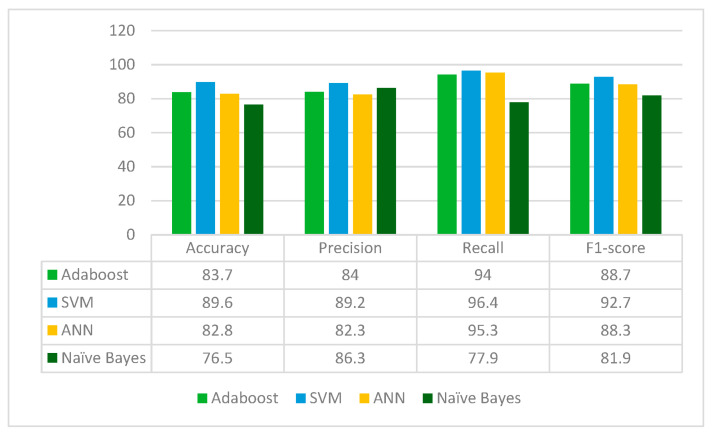
Results of ResNet152 Model for different classifiers.

ResNet152 is applied to the leukemia dataset to extract the deep-learned features. It extracts 2048 features from the leukemia images. The extracted 2048 features are fed to the Random Forest feature selection method, which selects 716 principal and main features from the feature pool. These selected features are further fed to four classifiers: Adaboost, SVM, ANN and Naïve Bayes. The results for each classifier are given in Figure 4.

From Figure 4, it can be deduced that ResNet152 is performs best with the SVM classifier whereas the Naïve Bayes classifier performs worst in terms of accuracy, precision, recall and F1-score. ResNet152 jointly with Random Forest feature selection method and SVM classifier gives an accuracy of 89.6%.

#### 3.3.2. Feature Extraction Using VGG16

VGG16 [26] is a pre-trained model consisting of sixteen convolutional layers followed by three dense layers. The convolutional layers are composed of 3 × 3 filters and the max-pooling layer with a 2 × 2 window is applied after every two convolutional layers. The depth of this model helps the network to learn complex features and patterns present in the leukemia images. The VGG16 model uses Rectified Linear Unit (ReLU) as the activation function, which adds non-linearity to the network. The VGG16 model is used as a feature extractor and tuned to obtain deep features from the images.

**Figure 5:**
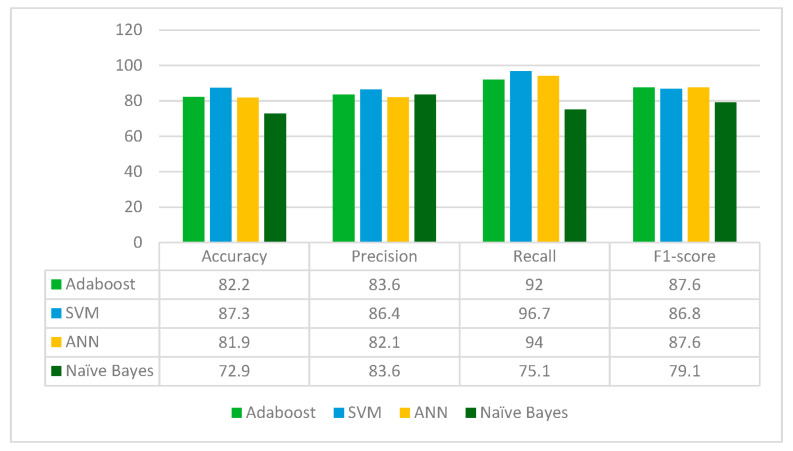
Results of the VGG16 model for different classifiers.

VGG16 is applied to the leukemia dataset to extract the deep-learned features. It extracts 512 features from the leukemia images. The extracted 512 features are fed to the Random Forest feature selection method, which selects 153 principal and main features from the feature pool. These selected features are further fed to four classifiers: Adaboost, SVM, ANN and Naïve Bayes. The results for each classifier are given in Figure 5.

From Figure 5, it can be inferred that VGG16 is the best performing with the SVM classifier whereas Naïve Bayes classifier is the worst performing in terms of accuracy, precision, recall and F1-score. VGG16 jointly with the Random Forest feature selection method and SVM classifier gives an accuracy of 87.3%.

#### 3.3.3. Feature Extraction Using DenseNet121

DenseNet-121 [27] is a deep CNN model that addresses the problems of vanishing gradients and information bottlenecks in deep neural networks. It comprises several dense blocks that contain numerous densely connected layers. Each layer in the model is connected to every other layer, which increases the information flow between the layers and hence facilitates feature reuse in the network. The denseNet121 model also consists of bottleneck layers and transition layers. The bottleneck layers comprise a 1 × 1 convolutional layer and a 3 × 3 convolutional layer. The 1 × 1 convolutional layer is responsible for reducing the number of input feature maps, reducing the computational cost, and enabling more compact representation. Transition layers are inserted between dense blocks to control the spatial dimensions and the number of channels in the network. They reduce the spatial resolution and compress the number of feature maps, thereby reducing the computational burden.

**Figure 6:**
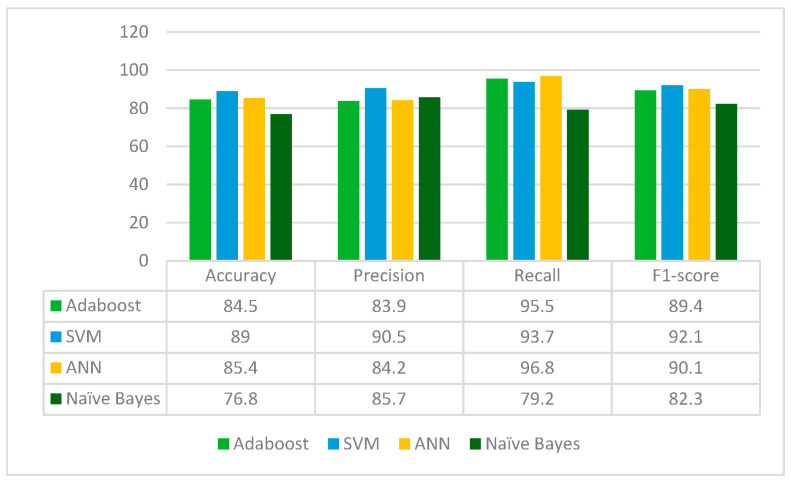
Results of DenseNet121 for different classifiers.

DenseNet121 is applied to the leukemia dataset to extract the deep-learned features. It extracts 1024 features from the leukemia images. The extracted 1024 features are fed to the Random Forest feature selection method, which selects 400 principal and main features from the feature pool. These selected features are further fed to four classifiers: Adaboost, SVM, ANN and Naïve Bayes. The results for each classifier are given in Figure 6.

From Figure 6, it can be concluded that DenseNet121 is the best performing with the SVM classifier whereas the Naïve Bayes classifier is the worst performing in terms of accuracy, precision, recall and F1-score. DenseNet121 jointly with the Random Forest feature selection method and SVM classifier gives an accuracy of 89%.

#### 3.3.4. Feature Extraction Using MobileNetV2

MobileNetV2 [28] is a convolutional neural network (CNN) architecture that comprises depthwise separable convolution, which splits the standard convolution operation into depthwise and pointwise convolutions. In the depthwise convolution, the same filter is applied to each input channel independently, reducing the computational cost. Pointwise convolutions then perform a 1 × 1 convolution to combine the output of depthwise convolutions across channels, allowing for richer feature interactions. The model also includes inverted residuals with linear bottlenecks, which expand the number of channels in the bottleneck layer and apply a depthwise separable convolution. All these features improve the efficiency of this model.

**Figure 7:**
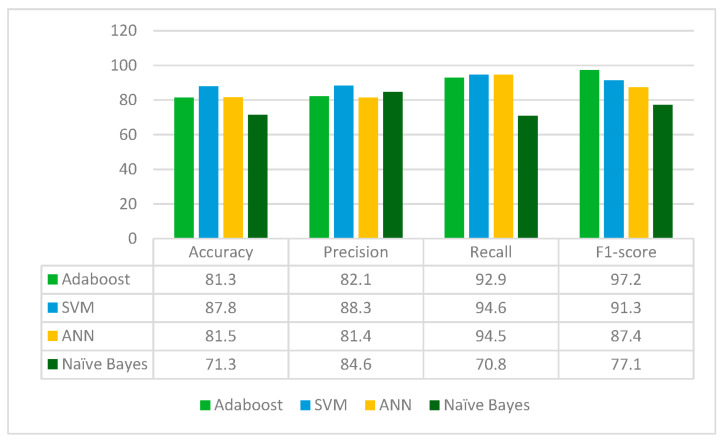
Results of MobileNetV2 for different classifiers.

MobileNetV2 is applied to the leukemia dataset to extract the deep learned features. It extracts 1280 features from the leukemia images. The extracted 1280 features are fed to Random Forest feature selection method, which selects 462 principal and main features from the feature pool. These selected features are further fed to four classifiers: Adaboost, SVM, ANN and Naïve Bayes. The results for each classifier are given in Figure 7.

From Figure 7, it can be inferred that MobileNetV2 is best performing with the SVM classifier whereas the Naïve Bayes classifier is the worst performing in terms of accuracy, precision, recall and F1-score. MobileNetV2 jointly with Random Forest feature selection method and SVM classifier gives the accuracy of 87.8%.

#### 3.3.5. Feature Extraction Using Inception V3

The InceptionV3 [29] model uses a multi-branch architecture with various filter sizes to capture information at different spatial scales. This allows the network to extract both local and global features effectively. It consists of Inception modules, which are the fundamental building blocks of the architecture. Each Inception module consists of multiple parallel convolutional branches with different filter sizes. These branches capture information at different scales and process it in parallel. Finally, the outputs of these branches are then concatenated along the channel dimension to form the module’s output. Inception V3 includes auxiliary classifiers at the intermediate stages of the network to encourage gradient flow and provide regularization.

**Figure 8:**
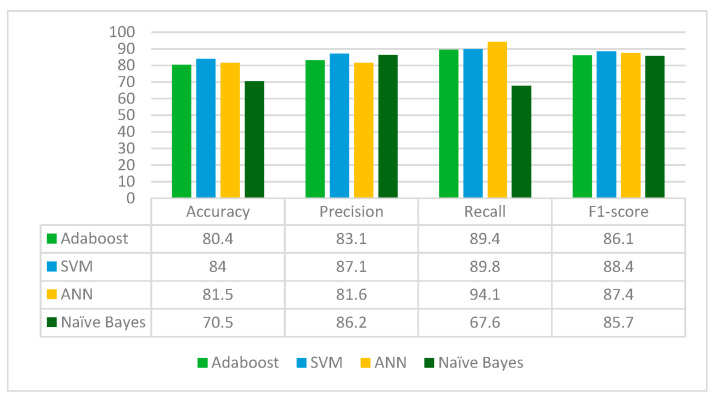
Results of InceptionV3 for different classifiers.

InceptionV3 is applied to a leukemia dataset to extract the deep learned features. It extracts 2048 features from the leukemia images. The extracted 2048 features are fed to Random Forest feature selection method, which selects 511 principal and main features from the feature pool. These selected features are further fed to four classifiers: Adaboost, SVM, ANN and Naïve Bayes. The results for each classifier are given in Figure 8.

From Figure 8, it can be deduced that InceptionV3 is the best performing with the SVM classifier whereas the Naïve Bayes classifier is the worst performing in terms of accuracy, precision, recall and F1-score. InceptionV3 jointly with Random Forest feature selection method and SVM classifier gives an accuracy of 84%.

#### 3.3.6. Feature Extraction Using EfficientNetB0

EfficientNetB0 [30] is the most minor and baseline variant of the EfficientNet models. It consists of a stack of convolutional layers with depth-wise separable convolutions, which reduce the number of parameters and computational cost. The architecture also employs a “compound scaling” technique that balances the model’s depth, width, and resolution to achieve better performance. The “B0” in EfficientNetB0 refers to the baseline version, with a width scaling factor of 1, depth scaling factor of 1, and an image resolution of 224 × 224 pixels. The scaling factor is used to increase or decrease the width and depth of the model while maintaining an optimum accuracy and computational efficiency.

**Figure 9:**
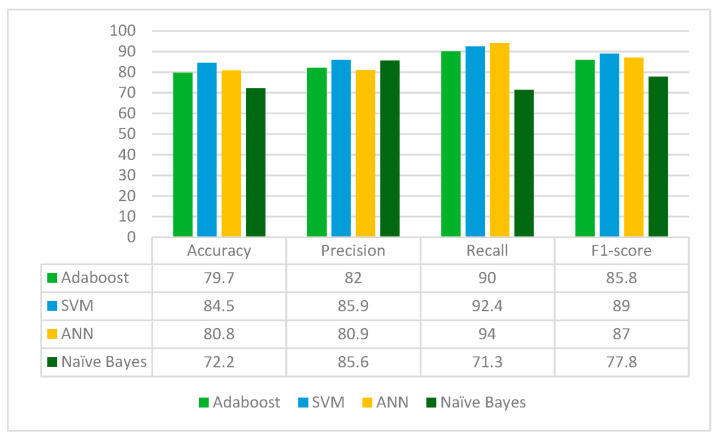
Results of EfficientNetB0 for different classifiers.

EfficientNetB0 is applied to the leukemia dataset to extract the deep learned features. It extracts 1280 features from the leukemia images. The extracted 1280 features are fed to the Random Forest feature selection method, which selects 292 principal and main features from the feature pool. These selected features are further fed to four classifiers: Adaboost, SVM, ANN and Naïve Bayes. The results for each classifier are given in Figure 9.

From Figure 9, it can be inferred that EfficientNetB0 is the best performing with the SVM classifier whereas the Naïve Bayes classifier is the worst performing in terms of accuracy, precision, recall and F1-score. EfficientNetB0 jointly with the Random Forest feature selection method and SVM classifier gives an accuracy of 84.5%.

#### 3.3.7. Feature Extraction Using ResNet50

The ResNet50 [31] model consists of fifty layers, out of which forty-eight are convolutional layers, and the other two are a max pool layer and an average pool layer. This model comprises stacked residual blocks. ResNet50 is a pre-trained DL model that can be used for feature extraction in numerous computer vision projects. These include classification of images, segmentation, and detection of objects in images. Feature extraction with ResNet50 uses the pre-trained weights of this model for the extraction of high-level features from input images.

**Figure 10:**
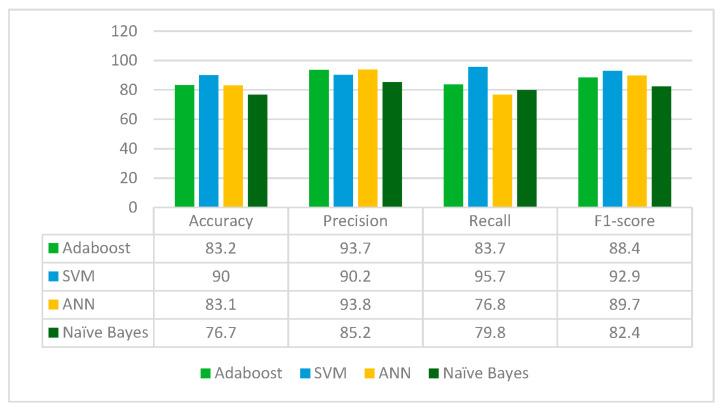
Results of ResNet50 for different classifiers.

ResNet50 is applied to the leukemia dataset to extract the deep learned features. It extracts 2048 features from the leukemia images. The extracted 2048 features are fed to Random Forest feature selection method, which selects 584 principal and main features from the feature pool. These selected features are further fed to four classifiers: Adaboost, SVM, ANN and Naïve Bayes. The results for each classifier are given in Figure 10.

From Figure 10, it can be concluded that ResNet50 is best performing with the SVM classifier whereas the Naïve Bayes classifier is the worst performing in terms of accuracy, precision, recall and F1-score. ResNet50 jointly with the Random Forest feature selection method and SVM classifier give an accuracy of 90%.

#### 3.3.8. Comparison of All Feature Extraction Techniques

From the above sections, it is clear that all transfer learning models perform best with the SVM classifier. So, for the remainder of the study, the SVM classifier will be used for the analysis of different feature selection methods. The results of all the feature extraction models are compared in Table 1 to discover the best feature extraction model.

From Table 1, it can be seen that ResNet50 performs best with the combination of SVM classifier and Random Forest feature selection method in terms of accuracy, precision, recall and F1-score. Hence, for further study of the feature selection method, the ResNet50 transfer learning model will be considered with the combination of SVM classifier. It is also clear from Table 1 that the shortest execution time is for the MobileNetV2 model and the longest execution time is for the VGG16 model. The ResNet50 model has the second-shortest execution time and also the best results in terms of error metrics.

### 3.4. Feature Selection Using Different Techniques

Here, the features extracted from the ResNet50 model are fed to three feature selection methods: ANOVA, Random Forest, and PCA. This process involves reducing the input variables for the model through the use of relevant data. Furthermore, the model eliminates the noise in the input data. Relevant features are chosen automatically for classification based on the problem. Essential features are either excluded or included without bringing any change to them. Feature selection cuts down on the noisy data and reduces the size of the input data.

In our work, 2048 features are extracted from the Resnet50 encoding network. From a pool of 2048 features, the top significant features are selected with the help of the three methods (ANOVA, Random Forest and PCA feature selection method). ANOVA selects the top 500 features, whereas the Random Forest method selects 584 features and PCA method selects 592 features. The performance of these three feature sets is analyzed by applying them to the SVM classifier and comparing their error metrics.

#### 3.4.1. Feature Selection with Analysis of Variance (ANOVA)

ANOVA feature selection [32,33,34] is a technique used in machine learning for selecting the most essential characteristics from the dataset. The ANOVA technique involves calculating the F-value for each feature, representing the degree to which the target variable’s variance can explain that feature’s variance. Features with higher F-values are considered more important, as they correlate strongly with the target variable. The ANOVA F-value is calculated by comparing the variance of a particular feature across different levels of the target variable.

Here, the F-statistic and associated *p*-value for each feature is initially calculated. These features are ranked based on their F-statistic values. Features with higher F-statistic values are more likely to be associated with significant differences in means between groups. Based on this, the top features are selected, for which a cutoff value for the F-statistic or *p*-value is set.

**Figure 11:**
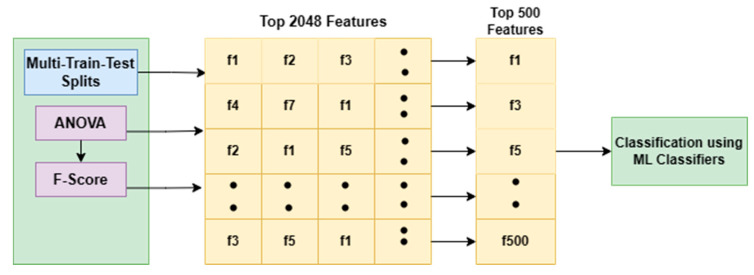
Feature selection using ANOVA.

Here in Figure 11, it can be seen that 2048 features are extracted from the ResNet50 encoding network. An ANOVA f-value is calculated for each feature by comparing the variance of a particular feature across different levels of the target variable. From 2048 features, the top 500 features with high F-value are shortlisted, indicating that their variation across different groups is significant. The selected top 500 features with the highest F-values are fed to the different classifiers for further classification.

The ANOVA feature selection is advantageous as it is a simple and efficient technique, which makes it suitable for large datasets with many features. However, it assumes that the features are typically distributed and that there is equal variance across groups, which may not always be the case in practice.

#### 3.4.2. Feature Selection with Random Forest

This method involves a technique helpful in selecting the best and relevant characteristics from the data using a Random Forest model [35]. This algorithm is an ensemble learning method which makes use of several decision trees to build a predictive model. For this, the Random Forest model is trained on the dataset using all the available features, i.e., 2048. Essential features are measured using the mean decrease impurity (MDI) of the feature. The MDI of a feature is calculated by measuring how much the impurity of the target variable is decreased when that feature is used in the decision trees. The features are ranked based on their importance scores. The higher the importance score, the more influential the feature is in predicting the target variable. Then, the top k = 584 features are selected based on their importance scores. The value of k is determined using the trial-and-error method. After that, a new Random Forest model is trained using only the chosen features.

**Figure 12:**
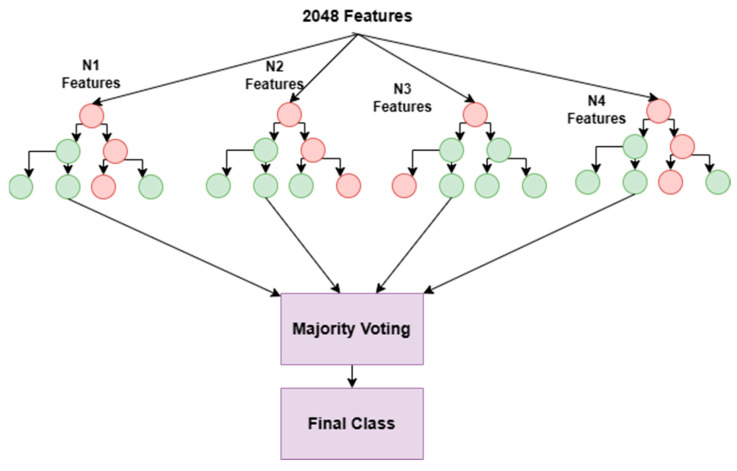
Feature selection using Random Forest.

Here as shown in Figure 12, 2048 features are extracted from the ResNet50 encoding network. From 2048 features, the top 584 features are selected that have large MDI. The selected top 584 features are fed to the different classifiers for further classification.

One of the advantages of Random Forest feature selection is that it can capture nonlinear relationships between the features and the target variable, which may not be possible with linear methods such as ANOVA. However, it can be computationally expensive for large datasets with many features, and the feature importance scores may be biased towards correlated features. Therefore, it is important to perform a careful evaluation of the selected features and their impact on the final model performance.

#### 3.4.3. Feature Selection with Principal Component Analysis (PCA)

PCA is a dimensionality reduction technique popularly used for feature selection in ML and data analysis. It helps in identification of the most essential features in a dataset based on their contribution to the principal components. In PCA, first the covariance matrix is computed, to describe the relationships between different features. Then the decomposition of the covariance matrix is performed to obtain the eigen values and the eigen vectors. The eigenvalues are sorted in the descending order and selection of the principal components is carried out. Furthermore, the feature importance is calculated and feature selection is carried out.

In this paper, PCA extracts 592 features from the pool of 2048 features obtained from the ResNet model.

#### 3.4.4. Comparison of Different Feature Selection Methods

**Figure 13:**
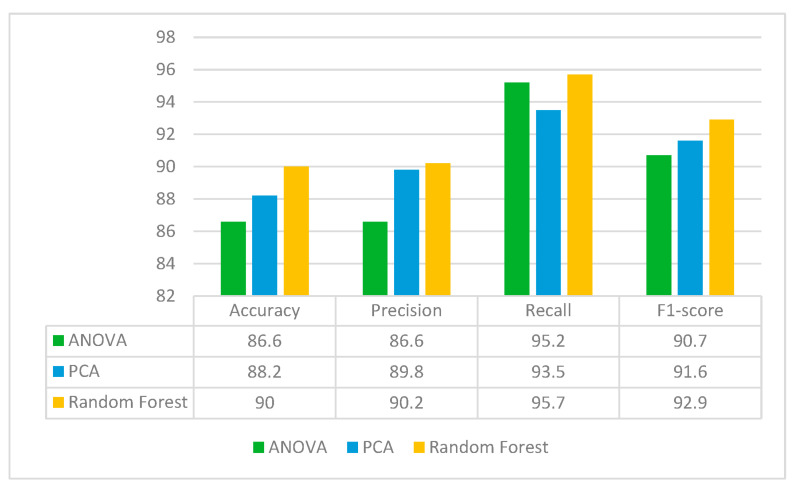
Performance metrics for different feature selection techniques with ResNet50 and SVM classifier.

It can be concluded from the previous section that the ResNet50 feature extraction model performed best out of seven models. Hence, ResNet50 is used for feature extraction in the proposed approach. Here, the feature selection method is used to reduce the dimensionality of the input data and improve model performance by selecting the most relevant features. Feature selection can enhance the interpretability of machine learning models by identifying the most important features that contribute to the predictions by removing irrelevant or redundant features. For this, three feature selection methods are used to best optimize the model. From ResNet50 TL, 2048 features were extracted. Out of 2048 features, the most important features are selected using three feature selection methods: ANOVA, PCA and Random Forest. ANOVA shortlisted 500 features from the 2048 features whereas PCA and Random Forest selected 592 and 584 features, respectively. The performance of these three feature selection methods is analyzed by classifying them with the SVM classifier. Figure 13 shows the performance of these feature selection methods in terms of accuracy, precision, recall and F1-score. From the figure, it can be seen that the classification results are best for the Random Forest feature selection method, with 90% accuracy. Hence, Random Forest is used further for feature selection in the proposed approach in which it extracts 584 important features from the pool of 2048 features.

### 3.5. Ablation Study

Table 2 presents the ablation study, which includes the four best-performing feature extraction models: ResNet152, DenseNet121, MobileNetV2, and ResNet50. Firstly, the feature selection model is not chosen, and the best performing machine learning classifier, SVM, is applied. A high accuracy of 84% is obtained by the DenseNet121 model with the SVM classifier, and no feature selection is applied. Furthermore, a deep learning classifier, ANN, is applied to the four feature extraction models without feature selection. For feature selection, Random Forest is applied to the four feature extraction models, and with the DL classifier, a high accuracy of 85.4% is obtained. However, the ML classifier’s highest accuracy of 90% is obtained by the SVM and ResNet50 models for the Random Forest feature selection model. Thus, the hybrid combination works based on the strengths of ResNet50 feature extraction, Random Forest feature selection, and an SVM classifier.

## 4. Comparison with State-of-the-Art Models

Table 3 compares different studies that have used various leukemia classification techniques and their corresponding results. Inbarani et al. [36] used an SVM technique to classify 368 images and attained an accuracy of 83%. Bigorra et al. [37] also used SVM for image classification, but a larger dataset of 916 images was used and they obtained an accuracy of 74%. Rawat et al. [38] used SVM to classify 130 images and achieved an accuracy of 87%. Ongun et al. [39] used the K-nearest neighbor (KNN) technique to classify 108 images and obtained an accuracy of 88%.

The proposed technique used ResNet50 as feature extraction, Random Forest as feature selection and SVM technique as classifier to classify 10,661 images and achieved the highest accuracy of 90%, and an F1-score of 0.929 among the referenced studies. For the same dataset, an F1-score of 0.918 was obtained by the Mixup Multi-Attention Multi-Task Learning Model (MMA-MTL) [40]. Furthermore, an accuracy of 86.6% was obtained by a hybrid model of AlexNet, Long Short-Term Memory (LSTM) and DenseNet for the same dataset [41]. Ding et al. [42] proposed an ensemble model for the same dataset, and an F1-score of 0.855 was obtained. For the dataset comprising 15,114 images, an accuracy of 78% was obtained [43]. Thus, it is clear that the author must choose an appropriate combination of techniques and algorithms to obtain the best results.

## 5. Conclusions and Future Work

Leukemia is a type of hematologic cancer that leads to an increase in abnormal white blood cells and, hence, a decrease in immunity. White blood cells are the prime component of the immune system. Acute lymphocytic leukemias are a type of blood cancer that was detected and classified in this paper using ResNet152, VGG16, DendeNet121, MobileNetV2, InceptionV3, EfficientNetB0, and ResNet50 feature extraction, and six machine learning algorithms. The algorithms used for classification are Naïve Bayes, Random Forest, K-nearest neighbor, ANN, Adaboost, and Support Vector Machine. It was found that the SVM classifier performed the best and achieved the highest accuracy of 90%. The novelty of this hybrid model lies in the specific combination and integration of ResNet-50 for feature extraction, Random Forest for feature selection, and Support Vector Machine for classification. This approach capitalizes on the strengths of each component to enhance the overall performance, interpretability, and generalization capabilities of the model in image classification tasks. By combining these three components, the hybrid model achieved improved classification accuracy compared to using each component individually.

In the future, an improvement in accuracy could be obtained by using other combinations of deep learning and machine learning algorithms. A hybrid dataset could also be created, and work can be carried out on that.

## Figures and Tables

**Table 1:** Comparison of all feature extraction techniques.

Model Name	Accuracy (%)	Precision (%)	Recall (%)	F1-Score (%)	Execution Time (s)
ResNet152	89.6	89.2	96.4	92.7	5898
VGG16	87.3	86.4	96.7	91.3	6084
DenseNet121	89	90.5	93.7	92.1	5232
MobileNetV2	87.8	88.3	94.6	91.3	4830
InceptionV3	84	87.1	89.8	88.4	5340
EfficientNetB0	84.5	85.9	92.4	89	5370
ResNet50	90	90.2	95.7	92.9	5220

**Table 2:** Ablation study.

Feature Extraction Model	Feature Selection (Random Forest)	Deep Learning Classifier (ANN)	Machine Learning Classifier (SVM)	Accuracy (%)
ResNet152	🗶	🗶	✔	83.2
DenseNet121	🗶	🗶	✔	84
MobileNetV2	🗶	🗶	✔	80.7
ResNet50	🗶	🗶	✔	82.7
ResNet152	🗶	✔	🗶	82.9
DenseNet121	🗶	✔	🗶	83.2
MobileNetV2	🗶	✔	🗶	80.1
ResNet50	🗶	✔	🗶	82.3
ResNet152	✔	✔	🗶	82.8
DenseNet121	✔	✔	🗶	85.4
MobileNetV2	✔	✔	🗶	81.5
ResNet50	✔	✔	🗶	83.1
ResNet152	✔	🗶	✔	89.6
DenseNet121	✔	🗶	✔	89
MobileNetV2	✔	🗶	✔	87.8
ResNet50	✔	🗶	✔	90

**Table 3:** Comparison with state-of-the-art models.

Reference	Number of Images	Technique	Results
Chaabane et al. [36]	368	SVM	Accuracy: 83%
Yang et al. [37]	916	SVM	Accuracy: 74%
Görtler et al. [38]	130	SVM	Accuracy: 87%
Inbarani et al. [39]	108	KNN	Accuracy: 88%
Abir et al. [40]	15,114	VGG19	Accuracy: 78%
Mathur et al. [41]	10,661	MMA-MTL	F1-score: 0.918
Shah et al. [42]	10,661	Alexnet + LSTM Dense	Accuracy: 86.6%
Ding et al. [43]	10,661	Ensemble Model	F1-score: 0.855
Proposed Deep Feature Selection based Approach	10,661	ResNet50 + SVM	Accuracy: 90% F1-score: 0.929

## Data Availability

Not appliable.
